# Tailored Sr–Bi_4_Ti_3_O_12_/Co_3_O_4_ S-scheme heterojunction for highly efficient solar-light photodegradation of crystal violet dye

**DOI:** 10.1039/d6ra03141b

**Published:** 2026-07-10

**Authors:** Rasmirekha Pattanaik, Suresh Kumar Dash, Rishabh Kamal

**Affiliations:** a Department of Chemistry, ITER, Siksha ‘O’ Anusandhan (Deemed to be University) Bhubaneswar Odisha 751030 India sureshdash@soa.ac.in

## Abstract

The development of efficient photocatalysts for wastewater treatment is crucial for mitigating organic pollution. In this work, a Sr-doped Bi_4_Ti_3_O_12_/Co_3_O_4_ S-scheme heterojunction was synthesized *via* a facile solid-state and precipitation method and evaluated for the solar-light-driven photodegradation of crystal violet (CV) dye. X-ray diffraction (XRD) confirmed the orthorhombic phase of Bi_4_Ti_3_O_12_ with successful Sr incorporation along with spinel Co_3_O_4_. FTIR spectra displayed characteristic metal oxygen vibrations, validating composite formation. UVDRS analysis revealed extended solar light absorption with a significantly reduced band gap of 1.6 eV compared to pristine Bi_4_Ti_3_O_12_ (2.8 eV). Photoluminescence studies showed suppressed charge recombination, indicating efficient charge carrier separation. Under solar -light irradiation, the Sr-doped Bi_4_Ti_3_O_12_/Co_3_O_4_ composite achieved ∼95% degradation of CV within 55 min, following pseudo-first-order kinetics (*k* = 0.035 min^−1^). Furthermore, the photocatalyst exhibited excellent stability and reusability over multiple cycles. These findings highlight the synergistic role of Sr doping and S-scheme heterojunction formation in enhancing photocatalytic performance, offering a promising pathway for the design of advanced materials for wastewater remediation.

## Abstract 2

The development of efficient photocatalysts for wastewater treatment is crucial for mitigating organic pollution.

## Graphical abstract

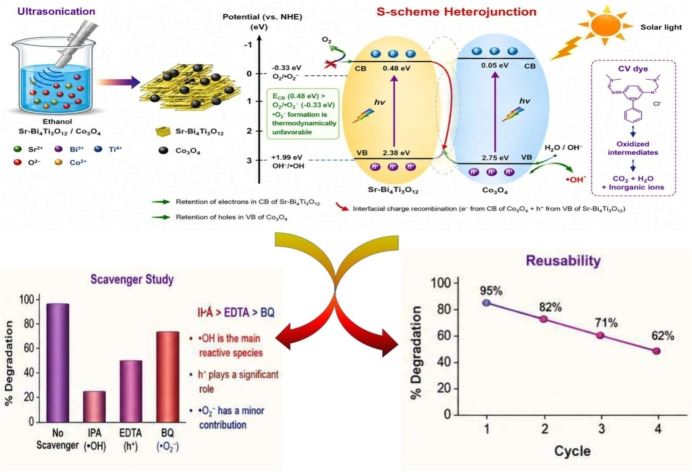

## 1. Introduction

The exponential advancement of industrial activities has significantly contributed to the intensification of environmental pollution, particularly in the form of dye-laden effluents discharged from textile, leather, cosmetic, paper, and printing industries.^1^ Among various classes of synthetic dyes used in these sectors, triphenylmethane dyes such as crystal violet (CV) have drawn increasing concern due to their persistent nature and high toxicity. CV is widely employed in dyeing, biological staining, and as a fungicide or antiseptic agent, yet its disposal into water bodies even at trace levels leads to severe ecological and health hazards. Its complex aromatic structure offers strong resistance to biodegradation and environmental breakdown, allowing it to persist in aquatic ecosystems and accumulate through the food chain. Prolonged exposure to CV has been associated with mutagenic, carcinogenic, and cytotoxic effects, making it a priority pollutant of global concern.^2–4^

Conventional wastewater treatment techniques such as coagulation–flocculation, activated sludge processes, adsorption on activated carbon, and membrane filtration, while effective in many contexts, often fall short in completely removing or degrading CV.^5^ These methods frequently result in phase transfer of pollutants without actual mineralization, generate secondary waste, or involve high operational costs. Consequently, there has been growing interest in developing alternative, sustainable, and cost-effective approaches that can achieve both high removal efficiency and environmental compatibility.^6,7^

In this context, solar-light-driven photocatalysis has emerged as a green and promising strategy for the degradation of organic dyes like CV. This method harnesses solar energy to activate semiconductor materials, initiating the generation of reactive oxygen species (ROS) such as hydroxyl and superoxide radicals capable of mineralizing pollutants into harmless byproducts like CO_2_ and H_2_O.^8^ However, the efficiency of single-component photocatalysts is often limited due to fast recombination of photogenerated electron–hole pairs, limited light absorption range, and poor surface reactivity. Hence, the design of advanced photocatalytic materials with enhanced charge separation and light harvesting capabilities is essential.^9,10^

Bismuth titanate (Bi_4_Ti_3_O_12_), a member of the Aurivillius oxide family, has garnered significant attention due to its layered perovskite-like structure, inherent ferroelectric properties, and solar-light responsiveness.^11^ Despite these advantages, pristine Bi_4_Ti_3_O_12_ suffers from relatively low surface area and rapid electron–hole recombination, which restrict its photocatalytic potential. One strategy to overcome these limitations involves the incorporation of heteroatoms or metal ions into its crystal lattice. Among such modifications, strontium (Sr^2+^) doping has proven effective in introducing oxygen vacancies and lattice distortions, improving charge carrier mobility, and extending solar-light absorption, thereby enhancing its photocatalytic performance.^12^

In parallel, constructing heterojunctions between semiconductors with complementary band alignments has become a powerful method to boost photocatalytic efficiency. Co_3_O_4_, a p-type transition metal oxide with a narrow bandgap (1.5–2.0 eV), exhibits strong redox potential, high photostability, and broad solar-light absorption.^13^ It's coupling with an n-type semiconductor like Sr-doped Bi_4_Ti_3_O_12_ can lead to the formation of a p–n heterojunction, facilitating efficient separation of charge carriers and enhancing surface reaction kinetics.^14^ In such heterojunctions, electrons and holes are directed along favourable paths, minimizing recombination and extending the lifetime of active species involved in dye degradation.

In recent years, several heterojunction-based photocatalysts have been investigated for the degradation of organic dyes and pollutants under solar light. For instance, Bi_4_Ti_3_O_12_/g-C_3_N_4_ composites have shown notable photocatalytic activity toward rhodamine B malachite green due to improved interfacial charge separation.^15^ Similarly, BiFeO_3_/Co_3_O_4_ heterostructures have exhibited enhanced degradation efficiency of malachite green and methylene blue, attributed to the synergistic p–n junction effects.^16^ Additionally, Bi_2_WO_6_/Co_3_O_4_ systems demonstrated high performance in removing tetracycline antibiotics under solar irradiation.^17^ While these systems offered valuable insights, most focused primarily on either photocatalysis or adsorption alone and often lacked synergistic enhancement from lattice doping. The simultaneous incorporation of Sr doping and heterojunction engineering remains underexplored, especially in the context of bifunctional removal of CV dye under solar light irradiation.

Building upon these gaps, the present study focuses on the development of a tailored Sr–Bi_4_Ti_3_O_12_/Co_3_O_4_ heterojunction that combines the advantages of Sr doping and S-scheme formation to create a bifunctional photocatalyst. This material is designed to perform efficiently under solar-light conditions, utilizing a dual mechanism that allows both strong adsorptive interaction in dark conditions and active photocatalytic degradation under light exposure. The synthesis strategy adopted here is simple, cost-effective, and scalable, enabling structural and optical tuning of the photocatalyst without the need for rare or toxic elements.

Comprehensive characterization using X-ray diffraction (XRD), field-emission scanning electron microscopy (FESEM), UV-Vis diffuse reflectance spectroscopy (DRS), photoluminescence (PL), and BET surface area analysis is carried out to elucidate the structural, morphological, and optical features of the synthesized material. Furthermore, the photocatalytic degradation of crystal violet under solar light is investigated, alongside dark adsorption studies, reusability tests, and kinetic modeling to evaluate the material's stability, mechanism, and practical applicability. The insights derived from this study aim to contribute to the rational design of multifunctional heterostructured materials for efficient dye remediation and broader environmental applications.

## 2. Experimental section

### 2.1 Materials and methods

All chemicals used in this study were of analytical grade and employed without further purification. Bismuth nitrate pentahydrate (Bi(NO_3_)_3_·5H_2_O), titanium dioxide (TiO_2_, anatase phase), and strontium nitrate (Sr(NO_3_)_2_) were used as precursors for the synthesis of Sr-doped Bi_4_Ti_3_O_12_. These reagents were purchased from Sigma-Aldrich and used as received. Cobalt nitrate hexahydrate (Co(NO_3_)_2_·6H_2_O) and sodium hydroxide (NaOH), both procured from Merck, were employed for the *in situ* growth of Co_3_O_4_ nanoparticles on the perovskite surface. Oxalic acid and ammonia solution were used as a chelating agent to promote homogeneous mixing during synthesis. Ethanol (99.9%) and deionized (DI) water were used throughout the experiments for washing and dispersion purposes. All solutions were freshly prepared using DI water. The model pollutant crystal violet (CV) dye was obtained from SRL Chemicals (India) and used without modification for the adsorption and photocatalytic degradation studies. All chemicals were stored in airtight containers in a dry environment and handled under ambient laboratory conditions unless otherwise specified.

### 2.2 Synthesis of the structured photocatalyst Sr–Bi_4_Ti_3_O_12_/Co_3_O_4_

**Fig. 1:**
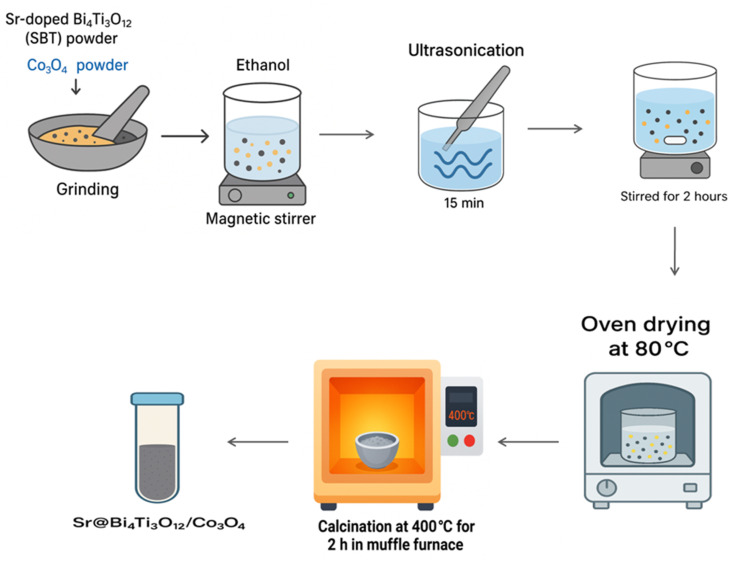
Schematic representation of the synthesis of Sr–Bi_4_Ti_3_O_12_/Co_3_O_4_ heterojunction.

4 wt% of appropriate Sr-doped Bi_4_Ti_3_O_12_ (SBT)^18^ and Co_3_O_4_ (ref. 19) powders were mixed in a desired weight ratio and ground thoroughly. The mixture was dispersed in ethanol, ultrasonicated for 15 minutes, and stirred for 2 hours to ensure uniform dispersion. After drying at 80 °C, the composite was calcined at 400 °C for 2 hours to enhance interfacial contact and form the heterojunction. The resulting Sr–Bi_4_Ti_3_O_12_/Co_3_O_4_ composite were then used for further characterization and dye removal studies. The pictorial representation of the procedure shown in Fig. 1.

### 2.3 Experimental set-up for photocatalytic tests

#### 2.3.1 Photocatalytic test

The photocatalytic degradation experiments of Crystal Violet (CV) dye were carried out using a batch reactor system under natural sunlight irradiation to evaluate the photocatalytic efficiency of the Sr–Bi_4_Ti_3_O_12_/Co_3_O_4_ nanocomposite. In a typical procedure, 25 mL of CV dye solution with a known initial concentration (ranging from 10 to 100 mg L^−1^) was taken in a 100 mL borosilicate glass beaker. A fixed dosage of photocatalyst (*e.g.*, 40 mg) was dispersed into the solution. The suspension was stirred in the dark for 30 minutes to establish adsorption–desorption equilibrium between the dye molecules and the surface of the catalyst. After equilibration, the reaction mixture was exposed to natural sunlight (typically between 10:00 AM and 2:00 PM) while maintaining continuous magnetic stirring to ensure uniform dispersion and light exposure. At regular time intervals (every 15 minutes), aliquots were withdrawn, filtered through Whatman Grade 42 filter paper, and analyzed using a UV-Vis spectrophotometer at 590 nm to determine the residual concentration of CV dye.^20^ The degradation efficiency (%) was calculated using the formula:1
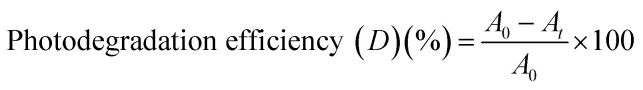
where *A*_0_ and *A*_*t*_ represent the initial and time-dependent absorbance of the dye. This systematic investigation enabled the evaluation and optimization of Sr–Bi_4_Ti_3_O_12_/Co_3_O_4_ photocatalysts for efficient CV dye degradation under solar irradiation.

#### 2.3.2 Kinetic, scavenger, and reusability test

To comprehensively evaluate the photocatalytic behaviour of Sr–Bi_4_Ti_3_O_12_/Co_3_O_4_ nanocomposites toward the degradation of Crystal Violet (CV) dye, kinetic, scavenger, and reusability studies were systematically conducted under sunlight irradiation.

The degradation kinetics was investigated by analyzing the concentration of CV dye at regular time intervals. The reaction followed a pseudo-first-order kinetic model, which was validated by plotting ln(*C*_0_/*C*) *versus* time (*t*).^21^ The apparent rate constant (*K*) was calculated using the equation:2
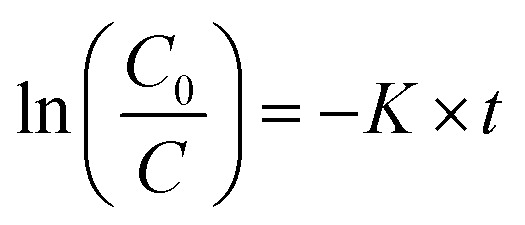
where *C*_0_ and *C* represent the initial and residual concentrations of CV dye (mg L^−1^), and *t* is the irradiation time (min). A linear correlation confirmed the pseudo-first-order kinetics, indicating that the degradation rate depends primarily on the dye concentration.

To identify the active species responsible for the photodegradation process, radical trapping experiments were carried out using specific scavengers. Isopropanol (IPA) was used to quench hydroxyl radicals (˙OH), benzoquinone (BQ) for superoxide radicals (˙O_2_^−^), and ammonium oxalate (AO) for photogenerated holes (h^+^). The photodegradation efficiency significantly decreased in the presence of these scavengers, confirming the crucial role of reactive oxygen species (ROS), especially ˙OH and ˙O_2_^−^, in the degradation mechanism. The results suggested that the photocatalytic activity proceeded *via* a multi-radical pathway typical of Z-scheme heterojunction systems.

The long-term stability and reusability of the Sr–Bi_4_Ti_3_O_12_/Co_3_O_4_ catalyst were examined over four consecutive photodegradation cycles. After each cycle, the photocatalyst was recovered by filtration, thoroughly washed with distilled water and ethanol to remove residual dye and adsorbed intermediates, and then dried at 80 °C. Only a slight decrease in efficiency was observed over successive cycles, indicating the high structural stability, chemical robustness, and recyclability of the nanocomposite for practical wastewater treatment applications.

### 2.4 Characterization analysis

The optical absorption behaviour and band gap energy of the Sr–Bi_4_Ti_3_O_12_/Co_3_O_4_ composite were assessed using a UV-Vis spectrophotometer (Jasco V-670). Functional groups and surface interactions were analyzed by Fourier Transform Infrared Spectroscopy (FT-IR, SHIMADZU IR-Affinity-1). Crystalline structure and phase composition were identified using X-ray Diffraction (XRD, BRUKER D8) with Cu Kα radiation (*λ* = 1.5406 Å), and the crystallite size was estimated using the Debye–Scherrer equation. Field Emission Scanning Electron Microscopy (FE-SEM, FEI Quanta FEG 250) was used to examine the surface morphology, while elemental distribution was confirmed through Energy-Dispersive X-ray Spectroscopy (EDAX). Transmission Electron Microscopy (TEM, JEOL JEM, and 100 kV) provided high-resolution images of the nanoscale structure. Photoluminescence (PL) spectroscopy was used to evaluate charge carrier recombination characteristics. The specific surface area and porosity were determined using Brunauer–Emmett–Teller (BET) analysis with nitrogen adsorption–desorption isotherms at 77 K (Quantachrome Nova 1000). Furthermore, X-ray Photoelectron Spectroscopy (XPS, PHI 5000 VersaProbe II) was employed to investigate the surface composition and oxidation states of the constituent elements in the composite.

## 3. Results and discussion

### 3.1 Structured photocatalyst characterization

**Fig. 2:**
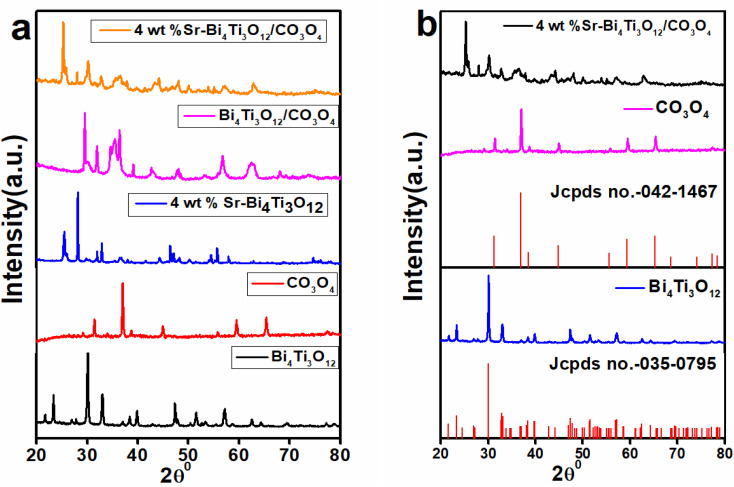
(a) XRD patterns, (b) JCPDS data confirmation of Bi_4_Ti_3_O_12_, Co_3_O_4_, 4 wt% Sr–Bi_4_Ti_3_O_12_ Bi_4_Ti_3_O_12_/Co_3_O_4_and 4 wt% Sr–Bi_4_Ti_3_O_12_/Co_3_O_4_ composites.

The X-ray diffraction (XRD) patterns of the synthesized samples, including pristine Bi_4_Ti_3_O_12_, Co_3_O_4_, Sr-doped Bi_4_Ti_3_O_12_, and their composites, are shown in Fig. 2a. The diffraction pattern of pure Bi_4_Ti_3_O_12_ exhibits sharp and well-resolved peaks, confirming the orthorhombic layered perovskite structure with high crystallinity. Characteristic peaks observed around 2*θ* = 28.30, 32.70, and 47.20 further validate the orthorhombic phase of Bi_4_Ti_3_O_12_.^22^ In contrast, Co_3_O_4_ displays diffraction peaks at 2*θ* = 19.00, 31.30, 36.80, 44.80, 59.40, and 65.20, indexed to the (111), (220), (311), (400), (511), and (440) planes of a cubic spinel structure (JCPDS card no. 42-1467), confirming its pure phase.^23^ For 4 wt% Sr-doped Bi_4_Ti_3_O_12_, the peak positions remain similar to pristine Bi_4_Ti_3_O_12_, indicating successful incorporation of Sr^2+^ ions into the Bi^3+^ lattice sites without altering the fundamental orthorhombic structure. Slight peak broadening and shifts suggest lattice distortion due to substitutional doping.^18^ The Bi_4_Ti_3_O_12_/Co_3_O_4_ composite reveal diffraction peaks belonging to both Bi_4_Ti_3_O_12_ and Co_3_O_4_ phases, confirming the formation of a binary heterostructure without impurity phases. Likewise, Sr–Bi_4_Ti_3_O_12_/Co_3_O_4_ exhibits all characteristic peaks of Sr-doped Bi_4_Ti_3_O_12_ and Co_3_O_4_, without additional reflections, suggesting a well-defined ternary composite. The observed peak broadening and slight shifts indicate enhanced structural disorder induced by both Sr doping and interfacial coupling with Co_3_O_4_. Fig. 2b shows the characteristic diffraction peaks of Bi_4_Ti_3_O_12_ (JCPDS no. 35-0795) and Co_3_O_4_ (JCPDS no. 42-1467). The Sr–Bi_4_Ti_3_O_12_/Co_3_O_4_ composite displays peaks of both phases without impurities, while slight peak broadening and intensity changes indicate structural modification and crystallite size variation due to Sr incorporation.

The crystallographic parameters, space groups, and average crystallite sizes were calculated using the Scherrer equation:3
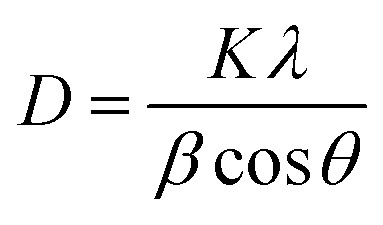
where *D* is the crystallite size, *K* is the shape factor (0.9), *λ* is the X-ray wavelength (1.5406 Å for Cu Kα), *β* is the full width at half maximum (FWHM, in radians), and *θ* is the Bragg angle. These results are summarized in Table 1, showing a clear reduction in crystallite size from 70 nm (Bi_4_Ti_3_O_12_) to 35 nm (Sr–Bi_4_Ti_3_O_12_/Co_3_O_4_) was observed, favouring higher surface area and more efficient charge transfer for photocatalysis.

**Table 1:** Crystallographic data of synthesized materials from XRD analysis

Sample	Phase composition	Crystal system	Space group	Crystallite size (nm)
Bi_4_Ti_3_O_12_ (pristine)	Bismuth titanate (Aurivillius)	Orthorhombic	*B*2*cb*	70
Co_3_O_4_ (pristine)	Cobalt oxide (spinel)	Cubic (spinel)	*Fd*3̄*m*	25
4 wt% Sr–Bi_4_Ti_3_O_12_	Sr-doped Bi_4_Ti_3_O_12_	Orthorhombic (doped)	*B*2*cb*	55
Bi_4_Ti_3_O_12_/Co_3_O_4_ composite	Composite (Aurivillius + spinel)	Mixed phases	*B*2*cb* + *Fd*3̄*m*	40
4 wt% Sr–Bi_4_Ti_3_O_12_/Co_3_O_4_ composite	Sr-doped Bi_4_Ti_3_O_12_ + Co_3_O_4_	Mixed phases	*B*2*cb* + *Fd*3̄*m*	35

**Fig. 3:**
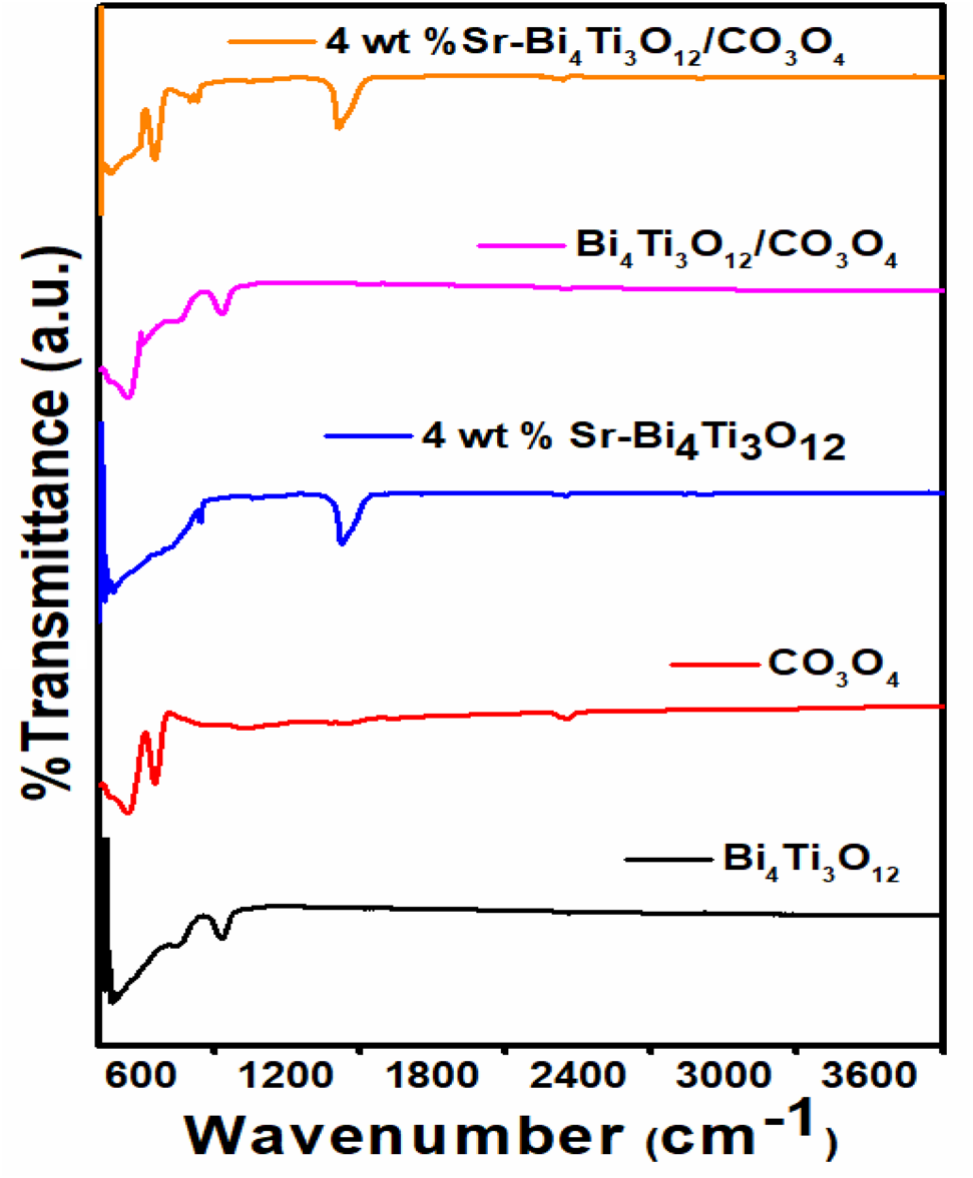
FTIR spectra showing Ti–O, Bi–O, and Co–O vibrations for Bi_4_Ti_3_O_12_, Co_3_O_4_, and their Sr-doped composites.

The FTIR spectra confirm the structural integrity and composition of all samples. Bi_4_Ti_3_O_12_ shows Ti–O and Bi–O vibrations in the 540–850 cm^−1^ range, typical of a perovskite structure.^24^ Co_3_O_4_ displays characteristic bands at ∼660 and ∼580 cm^−1^, corresponding to Co^3+^–O and Co^2+^–O bonds in the spinel phase.^25^ The 4 wt% Sr–Bi_4_Ti_3_O_12_ sample displays similar absorption bands to pure Bi_4_Ti_3_O_12_ but with slight shifts and intensity variations, suggesting successful incorporation of Sr^2+^ ions into the Bi_4_Ti_3_O_12_ lattice. This substitution may slightly alter the local symmetry and bond strength due to ionic size differences, leading to vibrational frequency shifts, especially in the 600–850 cm^−1^ region. The Bi_4_Ti_3_O_12_/Co_3_O_4_ composite exhibit both Ti–O and Co–O bands, suggesting effective heterojunction formation without secondary phases. The ternary Sr–Bi_4_Ti_3_O_12_/Co_3_O_4_ composite also shows overlapping vibrational features, confirming bonding integrity and interface interaction. Table 2 shows the FTIR peak positions and corresponding functional group, vibrational mode (Fig. 3).

**Table 2:** FTIR peak positions and corresponding functional group, vibrational mode

Sample	Wavenumber (cm^−1^)	Vibrational mode	Interpretation
Bi_4_Ti_3_O_12_	∼835	Ti–O stretching	Characteristic of TiO_6_ octahedra
	∼710	Bi–O stretching	Typical for perovskite Bi–O framework
Co_3_O_4_	∼660	Co^3+^–O stretching (octahedral)	Spinel structure vibration
	∼580	Co^3+^–O stretching (tetrahedral)	Confirms spinel phase
(4 wt%) Sr–Bi_4_Ti_3_O_12_	∼835	Ti–O	Sr^2+^ doping causes lattice distortion
	∼710 (shifted)	Bi–O (slightly shifted)	
Bi_4_Ti_3_O_12_/Co_3_O_4_	∼835, ∼710, 660, 580	Ti–O, Bi–O, Co–O	Composite formation with preserved parent phases
Sr–Bi_4_Ti_3_O_12_/Co_3_O_4_	∼835, ∼710, 660, 580	Ti–O, Bi–O, Co–O	Ternary system, confirms interaction and stability

### 3.2 Morphological analysis

**Fig. 4:**
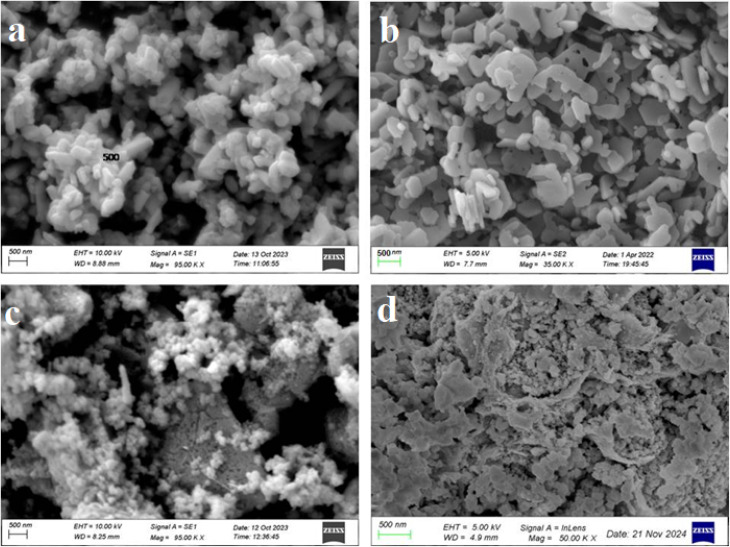
SEM images depict morphological variations of (a) Bi_4_Ti_3_O_12_, (b) Co_3_O_4_, (c) 4 wt% Sr–Bi_4_Ti_3_O_12_, and (d) 4 wt% Sr–Bi_4_Ti_3_O_12_/Co_3_O_4_ composites.

The morphology of each material and composite shown in Fig. 4. The SEM image of Bi_4_Ti_3_O_12_ reveals a typical layered platelet-like morphology, consistent with its Aurivillius-type crystalline structure in Fig. 4a.^26^ The grains are relatively smooth and compactly arranged, indicating a well-crystallized phase. In contrast, Co_3_O_4_ exhibits a flake-like and nanosheet rich morphology, offering a loosely packed architecture that enhances surface area and facilitates adsorption or redox activity in Fig. 4b.^27^ Upon Sr doping into Bi_4_Ti_3_O_12_, the morphology becomes more irregular and porous, with roughened surfaces and increased structural defects, suggesting lattice distortion and improved active site exposure in Fig. 4c. The final Sr–Bi_4_Ti_3_O_12_/Co_3_O_4_ composite shows a highly agglomerated and interconnected nanostructure, where Sr-doped Bi_4_Ti_3_O_12_ and Co_3_O_4_ are homogeneously mixed Fig. 4d. This architecture enhances interfacial contact, potentially benefiting photocatalytic or electrochemical performance through better charge transfer pathways.

**Fig. 5:**
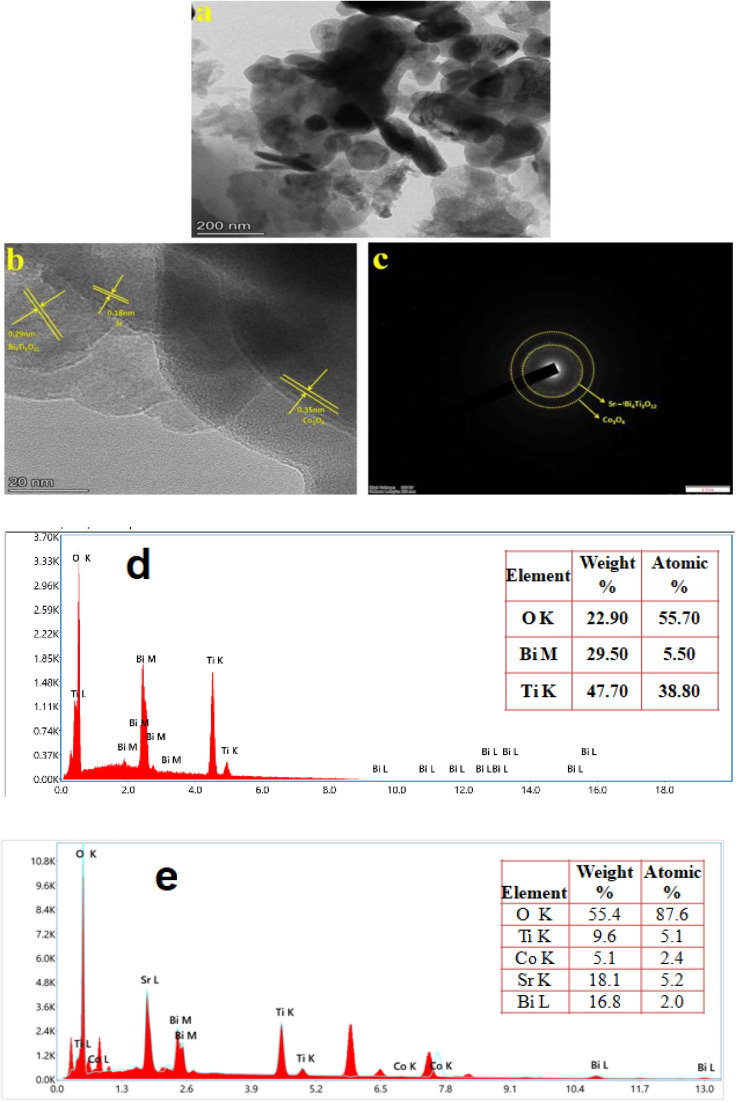
TEM images of (a) 4 wt% Sr–Bi_4_Ti_3_O_12_/Co_3_O_4_ heterojunction (b) HRTEM with lattice fringes of Bi_4_Ti_3_O_12_, Sr, and Co_3_O_4_, (c) SAED pattern confirming polycrystalline nature and EDS spectra of (d) Bi_4_Ti_3_O_12_ (e) 4 wt% Sr–Bi_4_Ti_3_O_12_/Co_3_O_4_ samples showing elemental composition and successful incorporation.

The TEM image of the Sr–Bi_4_Ti_3_O_12_/Co_3_O_4_ nanocomposite reveals a heterogeneous assembly of nanostructures with varying contrast, indicating the coexistence of multiple phases. Fig. 5a shows the overall morphology of the Sr–Bi_4_Ti_3_O_12_/Co_3_O_4_ heterojunction nanoparticles, where the particles exhibit irregular shapes and varying sizes, reflecting a well-dispersed nanocomposite structure. The dark contrast regions correspond to the denser Co_3_O_4_ phase, while the lighter contrast regions correspond to Bi_4_Ti_3_O_12_. Fig. 5b presents the HRTEM image highlighting the lattice fringes of different phases within the heterojunction. The measured interplanar spacings of 0.29 nm correspond to the (171) plane of Bi_4_Ti_3_O_12_ (ref. 28) and 0.18 nm correspond to Sr incorporation sites and 0.35 nm correspond to the (220) plane of Co_3_O_4_.^29^ The clear lattice fringes confirm the high crystallinity of the composite and the intimate interfacial contact between Sr–Bi_4_Ti_3_O_12_ and Co_3_O_4_, which is favourable for charge transfer. Fig. 5c shows the SAED pattern displaying distinct diffraction rings, confirming the polycrystalline nature of the Sr–Bi_4_Ti_3_O_12_/Co_3_O_4_ composite. The rings are indexed to Sr–Bi_4_Ti_3_O_12_ and Co_3_O_4_ crystal planes, further validating the coexistence of both phases in the heterojunction structure.

The EDS spectrum of the first sample shows the presence of Bi, Ti, and O with corresponding peaks and compositions, confirming the formation of Bi_4_T_3_O_12_ in Fig. 5d. In the second spectrum, additional peaks of Sr and Co are observed along with Bi, Ti, and O, verifying the successful incorporation of Sr and Co into the Bi_4_T_3_O_12_ matrix in Fig. 5e. The higher oxygen content reflects the oxide nature of the materials, while the reduced Bi content in the composite indicates partial substitution and loading with Sr and Co.

### 3.3 Surface area analysis

**Fig. 6:**
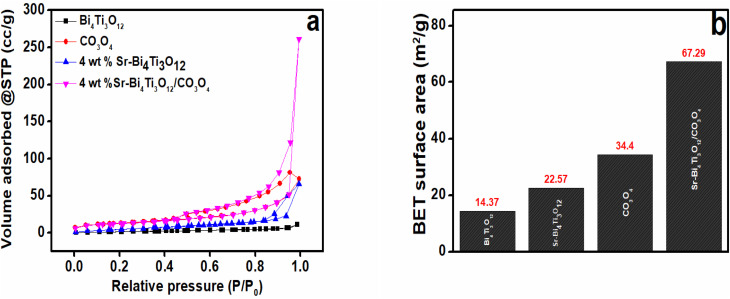
(a) N_2_ adsorption–desorption isotherms (b) BET surface area comparison of Bi_4_Ti_3_O_12_, Co_3_O_4_, 4 wt% Sr–Bi_4_Ti_3_O_12_, and 4 wt% Sr–Bi_4_Ti_3_O_12_/Co_3_O_4._

The nitrogen adsorption–desorption isotherms (Fig. 6a) and BET surface area analysis (Fig. 6b) provide crucial insights into the textural properties of Bi_4_Ti_3_O_12_, Co_3_O_4_, Sr-doped Bi_4_Ti_3_O_12_, and Sr–Bi_4_Ti_3_O_12_/Co_3_O_4_ nanocomposites. The isotherms exhibit typical type IV behaviour with H3-type hysteresis loops, indicative of mesoporous structures.^30^ Among the samples, the 4 wt% Sr–Bi_4_Ti_3_O_12_/Co_3_O_4_ composite shows the highest nitrogen uptake, suggesting a significantly enhanced porosity. This enhancement is quantitatively supported by BET surface area results, where the specific surface area increases from 14.37 m^2^ g^−1^ for Bi_4_Ti_3_O_12_ to 67.29 m^2^ g^−1^ for the Sr–Bi_4_Ti_3_O_12_/Co_3_O_4_ composite. The observed increase in surface area and pore volume upon Sr doping and heterojunction formation is expected to facilitate improved adsorption and catalytic performance, particularly in dye removal or photocatalytic applications.

### 3.4 Optical analysis

**Fig. 7:**
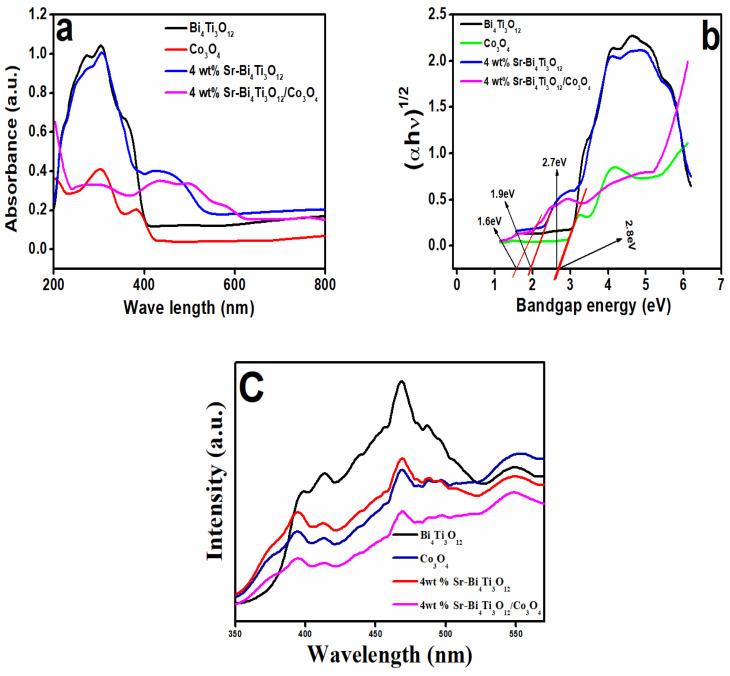
(a) UV-visible absorbance spectra (b) Tauc plots (*αℏν*)^1/2^*vs.* photon energy (c) PL spectra of Bi_4_Ti_3_O_12_, Co_3_O_4_, 4 wt% Sr–Bi_4_Ti_3_O_12_, and 4 wt% Sr–Bi_4_Ti_3_O_12_/Co_3_O_4_.

These Fig. 7a–c provide detailed optical and photophysical characterizations of Bi_4_Ti_3_O_12_, Co_3_O_4_, Sr–Bi_4_Ti_3_O_12_, and Sr–Bi_4_Ti_3_O_12_/Co_3_O_4_ composites. Fig. 7a presents the UV-Vis absorbance spectra of the materials across the 200–800 nm wavelength range. Bi_4_Ti_3_O_12_ shows strong absorption in the UV region with a sharp decline after ∼400 nm, indicating a wide bandgap. Co_3_O_4_ displays moderate absorbance into the visible region due to its narrower bandgap. Sr doping into Bi_4_Ti_3_O_12_ (blue line) slightly enhances the visible light absorption. Notably, the Sr–Bi_4_Ti_3_O_12_/Co_3_O_4_ composite (magenta) shows the broadest absorption extending well into the visible region, signifying improved light harvesting due to synergistic interaction and heterojunction formation. Fig. 7b shows the Tauc plots derived from the absorbance data, used to estimate the bandgap energies of the materials based on the Tauc relation.

4 (*αℏν*)^*η*^ = *A*(*ℏν* − *E*_g_)

where *α* is the absorption coefficient, *ℏν* is photon energy, *E*_g_ is the optical bandgap, *A* is a proportionality constant, and *n* = 1/2 for direct transition.

Bi_4_Ti_3_O_12_ has a bandgap of 2.8 eV, Co_3_O_4_ has 2.7 eV, and Sr-doped Bi_4_Ti_3_O_12_ shows a reduced bandgap of 1.9 eV. The composite Sr–Bi_4_Ti_3_O_12_/Co_3_O_4_ exhibits the lowest bandgap of 1.6 eV, indicating enhanced electronic interactions and superior light absorption efficiency, which is favourable for visible-light-driven photocatalysis.^31,32^Fig. 7c presents the photoluminescence (PL) spectra, which give insights into the recombination behaviour of photo-generated electron–hole pairs. Bi_4_Ti_3_O_12_ shows the highest PL intensity, indicating rapid charge recombination. Co_3_O_4_ and Sr-doped Bi_4_Ti_3_O_12_ show relatively lower PL intensity. The Sr–Bi_4_Ti_3_O_12_/Co_3_O_4_ composite exhibits the lowest PL intensity among all, suggesting significantly suppressed recombination and better charge separation, a crucial feature for efficient photocatalytic activity.

### 3.5 Elemental analysis

**Fig. 8:**
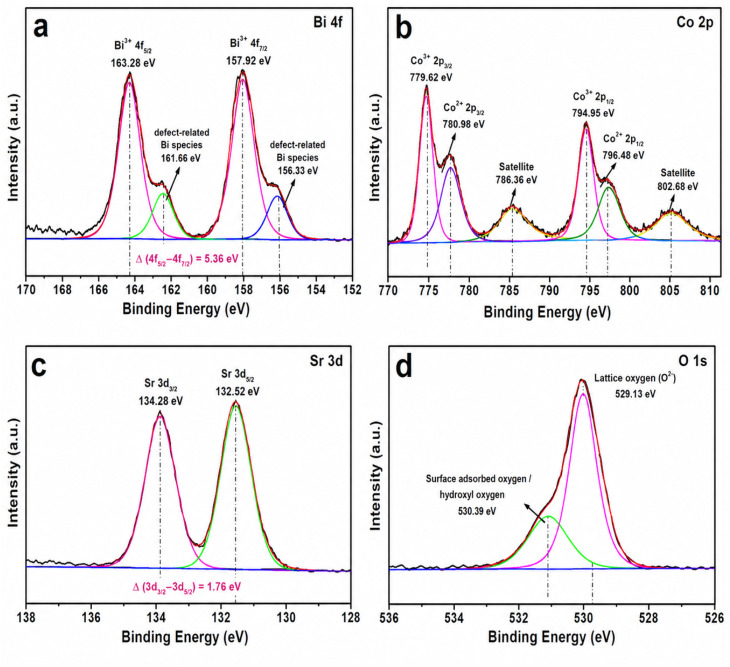
XPS spectra of 4 wt% Sr–Bi_4_Ti_3_O_12_/Co_3_O_4_ nanocomposite: (a) Bi 4f showing Bi^3+^ states, (b) Co 2p indicating the presence of both Co^2+^ and Co^3+^, (c) Sr 3d confirming Sr^2+^ incorporation, (d) O 1s is revealing lattice oxygen and surface-adsorbed oxygen species.

The X-ray Photoelectron Spectroscopy (XPS) analysis provides insight into the elemental composition and chemical states within the Sr–Bi_4_Ti_3_O_12_/Co_3_O_4_ nanocomposite. In the Bi 4f spectrum (Fig. 8a), distinct peaks at 163.12 eV and 157.87 eV are attributed to Bi^3+^ 4f_5/2_ and Bi^3+^ 4f_7/2_, respectively, confirming the presence of Bi^3+^. Additional peaks at 161.95 eV and 155.83 eV suggest the presence of sub-oxidized or metallic Bi species. The Co 2p spectrum (Fig. 8b) shows peaks at 779.73 eV and 780.75 eV corresponding to Co^3+^ and Co^2+^ 2p_3/2_, along with a satellite peak at 786.54 eV and a Co^2+^ 2p_1/2_ peak at 803.3 eV, indicating a mixed valence state of cobalt, characteristic of Co_3_O_4_. The Sr 3d spectrum (Fig. 8c) displays peaks at 133.54 eV and 132.82 eV, assigned to Sr^2+^ 3d_3/2_ and 3d_5/2_, confirming the successful incorporation of strontium. The O 1s spectrum (Fig. 8d) shows two peaks at 529.13 eV and 530.39 eV, corresponding to lattice oxygen and surface adsorbed oxygen or hydroxyl groups, respectively.^33^ The presence of mixed oxidation states and surface oxygen species suggests enhanced redox activity and potential for improved photocatalytic performance. Furthermore, the slight positive shift of Bi 4f and Sr 3d peaks alongside the negative shift in Co 2p peaks indicates interfacial charge redistribution between Sr–Bi_4_Ti_3_O_12_ and Co_3_O_4_. Such binding energy shifts confirm electron transfer from Sr–Bi_4_Ti_3_O_12_ to Co_3_O_4_ until Fermi level equilibration, which is a hallmark of S-scheme heterojunction formation. In this mechanism, photogenerated electrons in the CB of Co_3_O_4_ recombine with holes in the VB of Sr–Bi_4_Ti_3_O_12,_ retaining the strongly reducing electrons in Sr–Bi_4_Ti_3_O_12_ and the oxidative holes in Co_3_O_4_, thereby maximizing redox potential and enhancing photocatalytic efficiency.

## 4. Photocatalytic activity of crystal violet varying with different parameters

**Fig. 9:**
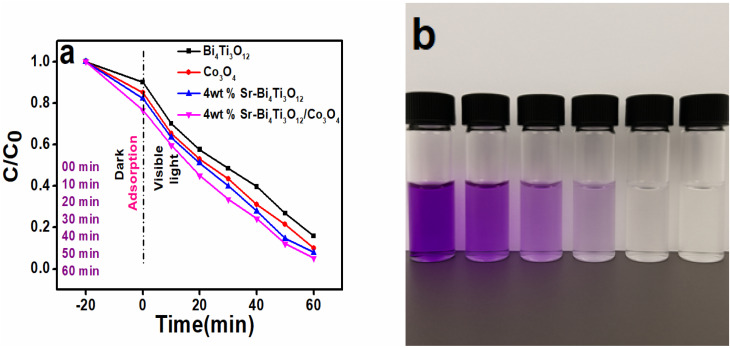
Photocatalytic degradation of crystal violet (40 ppm) under sunlight: (a) degradation profile showing enhanced activity of photocatalysts, (b) gradual fading of the purple solution with irradiation time.

The photocatalytic degradation of crystal violet (CV) was studied at a constant concentration of 40 ppm (40 mg L^−1^) using a 200 mL aqueous solution and a catalyst dose of 0.5 g L^−1^. The catalyst was dispersed into the dye solution, ultrasonicated for 5–10 minutes to achieve uniform suspension, and then magnetically stirred in the dark for 20 minutes to establish adsorption–desorption equilibrium. The clarified aliquot was analyzed at the characteristic absorption maximum of CV (*λ*_max_ = 590 nm) to determine the initial concentration (*C*_0_). The photocatalytic experiments were conducted under natural sunlight on clear-sky days, typically between 11:00 AM and 2:00 PM. IST, when the solar irradiance averaged 800–950 W m^−2^ (AM 1.5). The reactor vessel was placed outdoors at a 30–35° tilt angle, continuously stirred to prevent sedimentation, and maintained at 25 ± 2 °C using a circulating water bath. To closely follow the degradation process, 3–5 mL aliquots were collected every 5 minutes, centrifuged or filtered through 0.22–0.45 µm membranes, and the supernatant was analyzed by UV-Vis spectroscopy at 590 nm.^34^ The normalized concentration values (*C*/*C*_0_) were then plotted as a function of irradiation time, which revealed the fastest degradation for the Sr–Bi_4_Ti_3_O_12_/Co_3_O_4_ heterojunction, confirming its superior sunlight-driven photocatalytic activity compared to individual components in Fig. 9a. The series of vials shown in the image visually demonstrates this degradation process, where the intense purple colour of the initial crystal violet solution gradually fades with irradiation time in Fig. 9b, ultimately leading to a nearly colourless solution highlighting the efficiency of the catalyst in removing dye pollutants.

### 4.1 Effect of pH

**Fig. 10:**
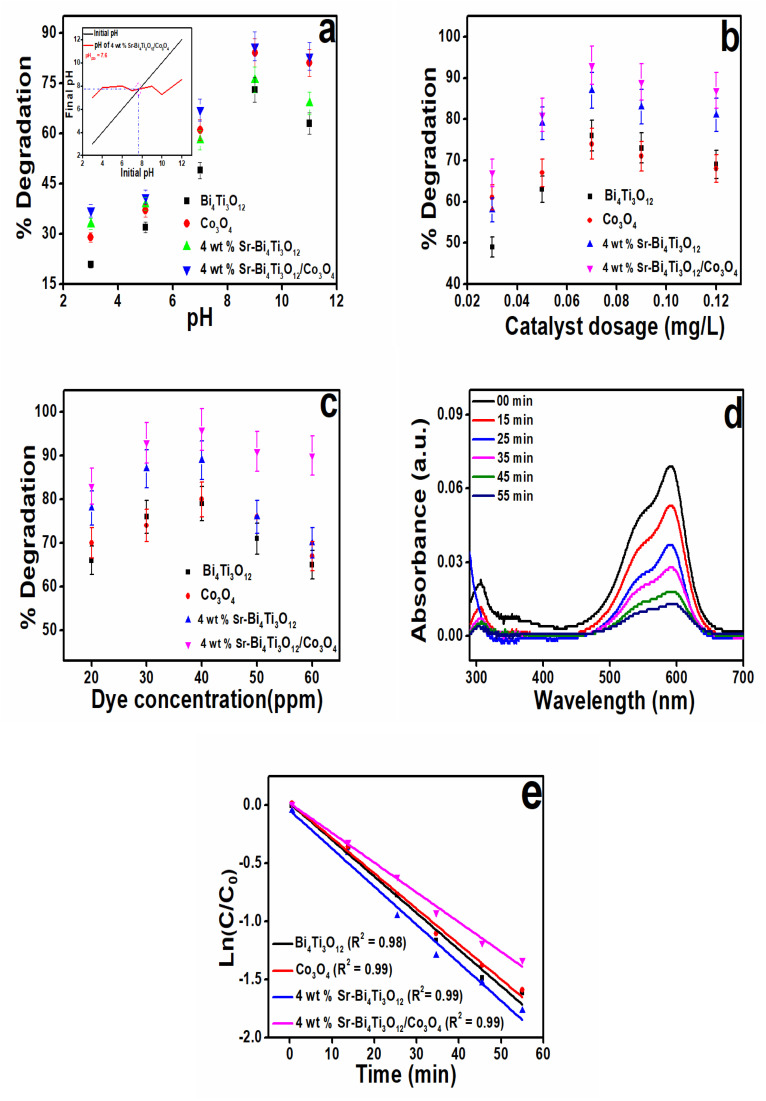
(a) Effect of pH, (b) catalyst dosage, (c) dye concentration on photocatalytic degradation efficiency, (d) UV-Vis absorption spectra showing degradation over time, (e) kinetic analysis of the degradation process.

The Fig. 10a illustrates the influence of pH on the percentage degradation of Crystal Violet (CV) dye using different photocatalysts: Bi_4_Ti_3_O_12_, Co_3_O_4_, Sr–Bi_4_Ti_3_O_12_, and the composite Sr–Bi_4_Ti_3_O_12_/Co_3_O_4_. It is evident that the degradation efficiency increases with rising pH for all samples, with the highest performance observed at pH 9. Among the tested materials, the Sr–Bi_4_Ti_3_O_12_/Co_3_O_4_ heterojunction consistently shows the highest degradation efficiency across all pH values, achieving nearly 87% degradation at pH 9. This enhanced activity is attributed to the synergistic effect between Sr-doping and Co_3_O_4_ coupling, which improves charge separation and surface interaction with the dye molecules. In contrast, Bi_4_Ti_3_O_12_ alone shows the lowest degradation, particularly in acidic conditions. The inset of the graph presents the relationship between initial and final pH for Sr–Bi_4_Ti_3_O_12_/Co_3_O_4_, highlighting a point of zero charge (pH_pzc_) at approximately 7.6. At pH values above this point, the photocatalyst surface becomes negatively charged, promoting stronger electrostatic attraction with the cationic CV dye, thereby enhancing degradation. This analysis indicates that alkaline conditions (pH > 7.6) are favourable for efficient dye removal using the Sr–Bi_4_Ti_3_O_12_/Co_3_O_4_ composite.

### 4.2 Effect of catalyst dosage

Fig. 10b shows the effect of catalyst dosage (mg L^−1^) on the percentage degradation of dye. With increasing catalyst dosage from 0.03 mg L^−1^ to 0.12 mg L^−1^, the degradation efficiency increases for all materials due to the availability of more active sites. However, Sr–Bi_4_Ti_3_O_12_/Co_3_O_4_ consistently exhibits the highest degradation efficiency, reaching around 95% at 0.08 mg L^−1^, outperforming Sr–Bi_4_Ti_3_O_12_, Co_3_O_4_, and Bi_4_Ti_3_O_12_. This superior performance is attributed to the heterojunction formation and Sr doping, which enhances solar light absorption and suppresses electron–hole recombination.

### 4.3 Effect of dye concentration

Fig. 10c illustrates the effect of varying dye concentration (ppm) on degradation performance. As dye concentration increases from 20 to 60 ppm, a general decline in degradation efficiency is observed across all catalysts. This is due to the higher dye load reducing light penetration and overwhelming the reactive species. Nonetheless, Sr–Bi_4_Ti_3_O_12_/Co_3_O_4_ still maintains the highest degradation percentage (∼95% at 30–40 ppm), indicating its strong photocatalytic potential even under higher pollutant loads. Sr–Bi_4_Ti_3_O_12_ also performs better than the individual components, but slightly less efficiently than the composite.

### 4.4 Effect of irradiation time

Fig. 10d illustrates the UV-Vis absorption spectra of the dye solution at various time intervals (0 to 55 minutes) in the presence of a catalyst, most likely the Sr–Bi_4_Ti_3_O_12_/Co_3_O_4_ composite. A prominent absorption peak around 590–600 nm, characteristic of the dye, gradually decreases in intensity with time, indicating the dye's degradation. The continuous decline in absorbance signifies efficient photocatalytic activity over time, with minimal residual dye at 55 minutes, demonstrating effective photodegradation.

### 4.5 Kinetic study

Fig. 10e presents the kinetic analysis of the photocatalytic degradation process, plotted as ln(*C*/*C*_0_) *versus* time. The linearity of the plots for all four catalysts confirms that the degradation follows pseudo-first-order kinetics. Among them, Sr–Bi_4_Ti_3_O_12_/Co_3_O_4_ exhibits the steepest slope, indicating the fastest degradation rate and highest rate constant. Sr–Bi_4_Ti_3_O_12_ also shows better performance than the individual Bi_4_Ti_3_O_12_ and Co_3_O_4_, reinforcing the synergistic effect of Sr doping and heterojunction formation. The *R*^2^ values (close to 0.99) confirm excellent fitting to the kinetic model. A comparative analysis of our Sr–Bi_4_Ti_3_O_12_/Co_3_O_4_ S-scheme heterojunction with other reported photocatalysts (Table 3) demonstrates its superior solar-light-driven CV degradation efficiency and competitive reaction time.

**Table 3:** Comparison of photocatalytic performance in UV/solar/visible-light degradation of crystal violet

No.	Photocatalyst	Removal efficiency (%)	Reaction time (min)	Light source	Reference
1	g-C_3_N_4_–CuO–ZnO	99.26	180	Sunlight	35
2	WO_3_/Ag/g-C_3_N_4_	95	120	Visible light	36
3	MoS_2_/BiVO_4_/g-C_3_N_4_	96	30	Sunlight	37
4	g-C_3_N_4_/FeTiO_3_/MnFe_2_O_4_	99.05	100	Visible light	38
5	ZnO/CuO/Ag_2_O	99.05	105	Solar light	39
6	MoS_2_–NiO–CuO	95	80	UV-visible light	40
7	30 wt% SrTiO_3_/BiOI	92.5	30	Solar light	41
8	Sr–Bi_4_Ti_3_O_12_/Co_3_O_4_	94.62%	55	Sunlight	This work

### 4.6 Reusability, and structural stability analysis

**Fig. 11:**
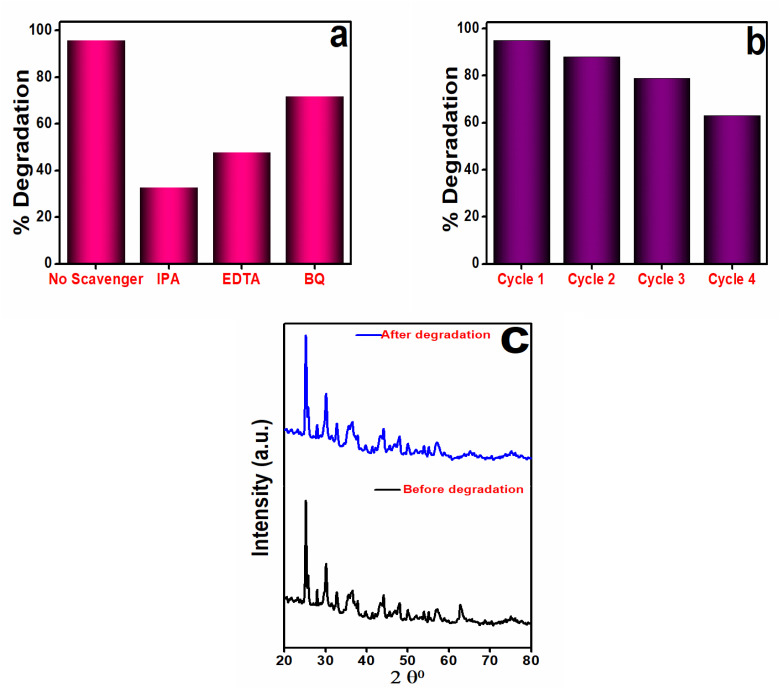
(a) Scavenger effect on degradation efficiency, (b) reusability over four cycles, (c) XRD patterns showing structural stability before and after degradation.

The provided graphs collectively illustrate the photocatalytic efficiency, reactive species involvement, and stability of the synthesized photocatalyst. Fig. 11a presents the scavenger study, where the degradation efficiency significantly decreases in the presence of IPA, EDTA, and BQ compared to the system without scavengers. The strongest inhibition by IPA confirms that hydroxyl radicals (˙OH) are the primary reactive species responsible for crystal violet degradation. Importantly, EDTA causes a more significant suppression than BQ, indicating that photogenerated holes (h^+^) contribute more substantially than superoxide radicals (˙O_2_^−^). Since the conduction band potential of Sr–Bi_4_Ti_3_O_12_ (+0.48 eV *vs.* NHE) is more positive than the O_2_/˙O_2_^−^ reduction potential (−0.33 eV *vs.* NHE), the formation of ˙O_2_^−^ is thermodynamically unfavorable. Therefore, the photocatalytic degradation process is mainly governed by ˙OH radicals and direct hole oxidation, while ˙O_2_^−^ plays only a limited secondary role. Fig. 11b shows that degradation efficiency decreases from about 95% in cycle 1 to around 62% in cycle 4, indicating some reduction in activity due to catalyst loss or surface fouling during repeated use; however, the photocatalyst still retains reasonable reusability. Fig. 11c presents the XRD patterns before and after degradation, where the nearly unchanged diffraction peaks confirm that the crystal structure remains intact, demonstrating good structural stability. Overall, these results confirm the photocatalyst's effectiveness, durability, and practical potential for long-term solar-driven wastewater treatment.

**Fig. 12:**
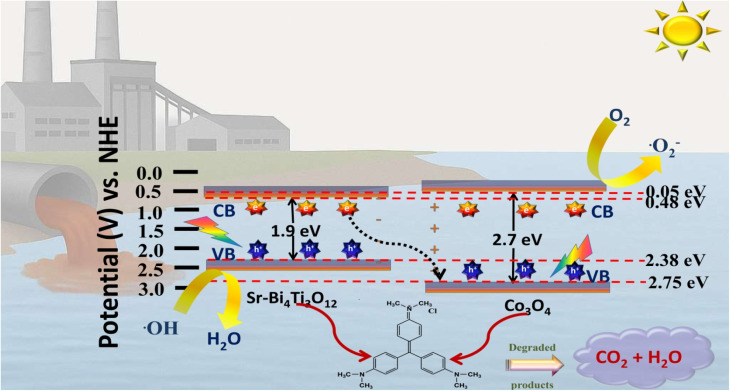
S-scheme mechanism for crystal violet degradation over Sr–Bi_4_Ti_3_O_12_/Co_3_O_4_ under solar light.

The Fig. 12 illustrates the S-scheme photocatalytic degradation mechanism of crystal violet (CV) dye using a Sr-doped Bi_4_Ti_3_O_12_ (Sr–Bi_4_Ti_3_O_12_)/Co_3_O_4_ heterojunction under solar-light irradiation. When exposed to sunlight, both Sr–Bi_4_Ti_3_O_12_ (*E*_g_ ≈ 1.9 eV) and Co_3_O_4_ (*E*_g_ ≈ 2.7 eV) absorb photons, exciting electrons from their valence band (VB) to conduction band (CB) (eqn (7) and (8)). The band-edge positions can be calculated using the following equations:5
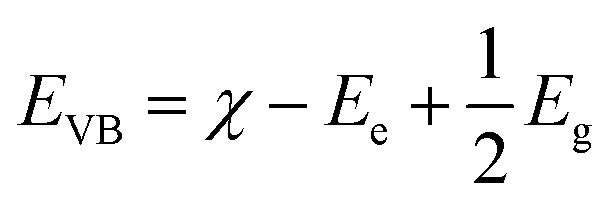

6 *E*_CB_ = *E*_VB_ − *E*_g_

where *E*_VB_ and *E*_CB_ are the valence and conduction band edge potentials (*vs.* NHE), *χ* is the absolute electronegativity of the semiconductor (eV), *E*_e_ is the energy of free electrons on the hydrogen scale (≈4.5 eV), and *E*_g_ is the band gap energy (eV).

In the S-scheme heterojunction, photogenerated electrons in the conduction band of Co_3_O_4_ recombine with holes in the valence band of Sr–Bi_4_Ti_3_O_12_ (eqn (9)), eliminating low-energy charge carriers while preserving highly oxidative holes in the VB of Co_3_O_4_ and electrons in the CB of Sr–Bi_4_Ti_3_O_12_. However, because the CB potential of Sr–Bi_4_Ti_3_O_12_ is insufficient for efficient reduction of O_2_ to ˙O_2_^−^, the superoxide pathway is thermodynamically suppressed. Instead, the dominant degradation mechanism proceeds mainly through direct oxidation by holes and hydroxyl radical generation *via* oxidation of H_2_O/OH^−^ by holes in the VB of Co_3_O_4_ (eqn (10) and (11)). These highly reactive holes and ˙OH radicals attack CV molecules, breaking their chromophoric structure, degrading them into smaller intermediates, and ultimately mineralizing them into CO_2_, H_2_O, and inorganic ions (eqn (12) and (13)). Thus, the enhanced photocatalytic efficiency of the Sr–Bi_4_Ti_3_O_12_/Co_3_O_4_ heterojunction is primarily attributed to efficient charge separation, strong hole oxidation, and hydroxyl radical generation, while the contribution of ˙O_2_^−^ radicals is minimal.

7 Sr–Bi_4_Ti_3_O_12_ + *ℏν* → e^−^(CB_SrBTO_) + h^+^(VB_SrBTO_)

8 Co_3_O_4_ + *ℏν* → e^−^(CB_Co_3_O_4__) + h^+^(VB_Co_3_O_4__)

9 e^−^(CB_Co_3_O_4__) + h^+^(VB_SrBTO_) → heat

10 h^+^(VB_Co_3_O_4__) + H_2_O → ˙OH + H^+^

11 h^+^(VB_Co_3_O_4__) + OH^−^ → ˙OH

12 h^+^(VB_Co_3_O_4__) + CV → oxidized intermediates

13 CV + ˙OH + h^+^ → CO_2_ + H_2_O + Cl^−^ + NO_3_^−^

### 4.7 Intermediate prediction

**Fig. 13:**
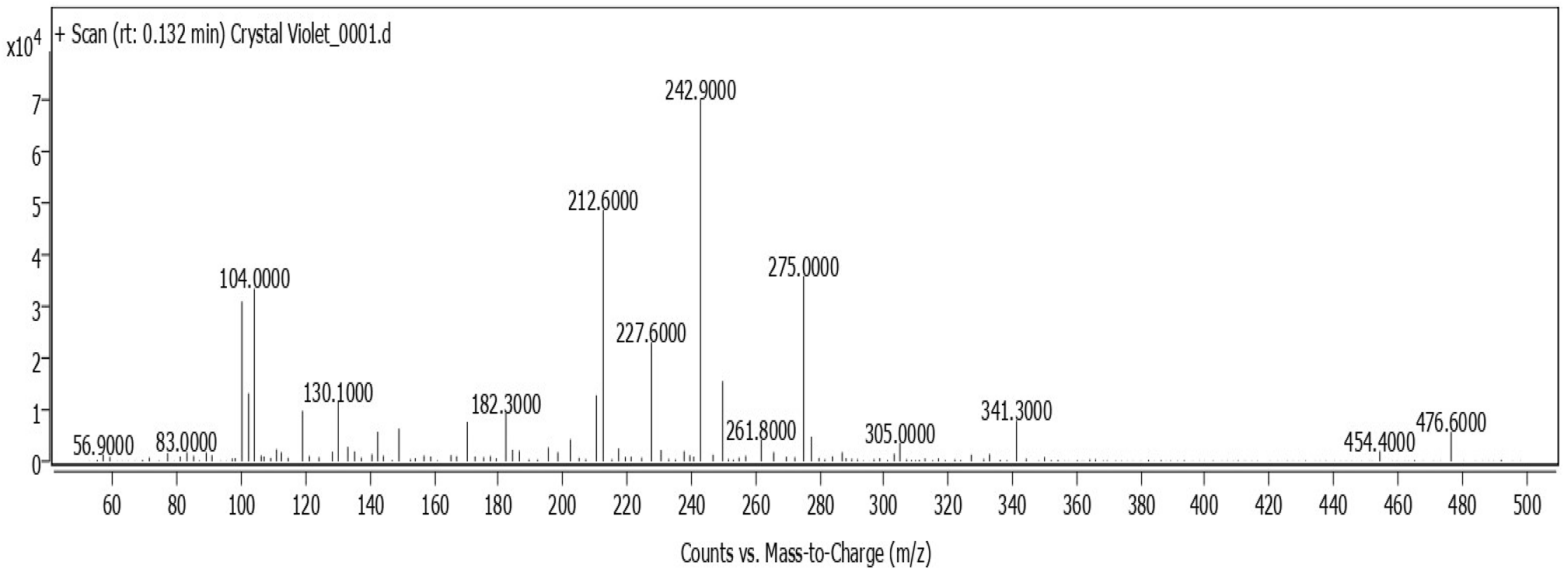
HRMS spectra of after degradation of crystal violet using Sr–Bi_4_Ti_3_O_12_/Co_3_O_4_ composite material.

**Fig. 14:**
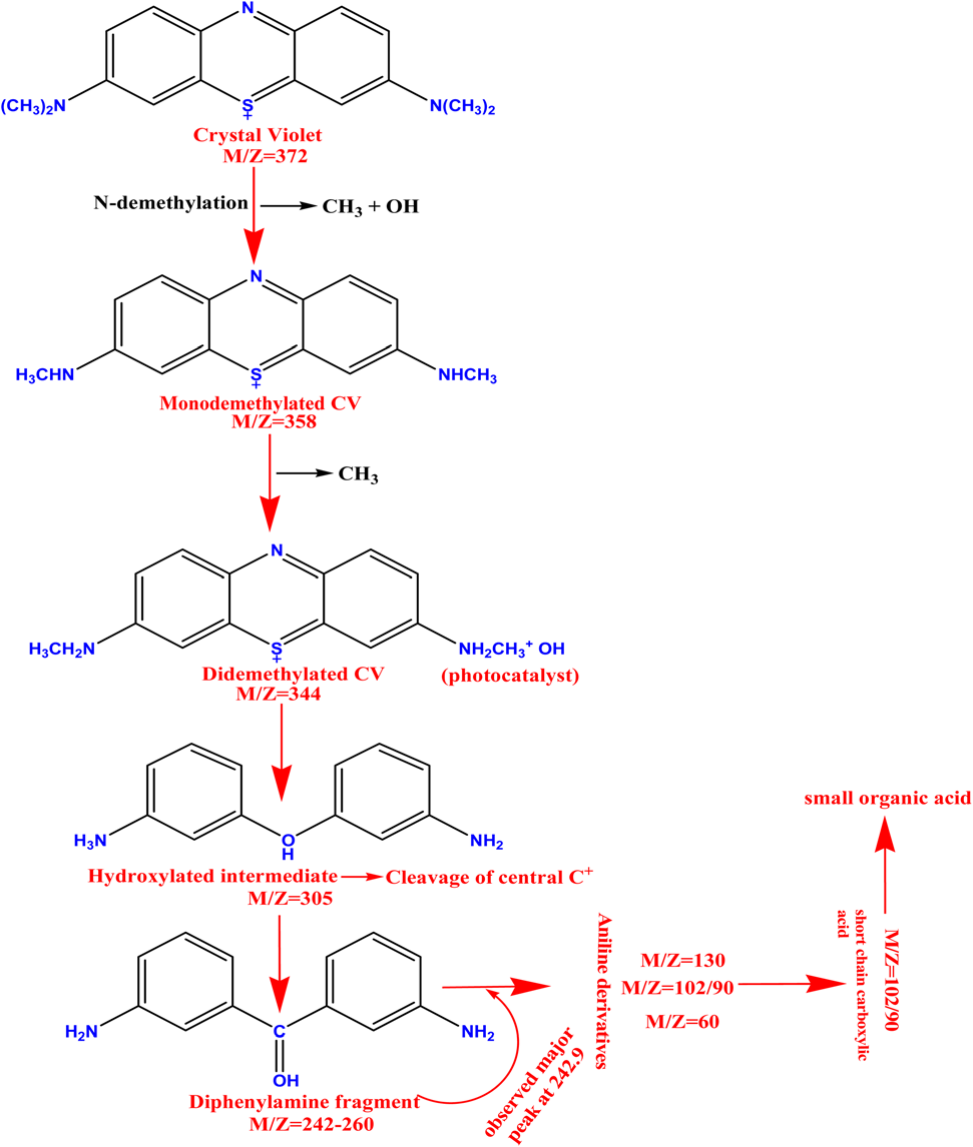
Proposed mechanism of degradation of crystal violet dye.

The HRMS spectrum recorded after photocatalytic degradation of crystal violet clearly demonstrates the structural disintegration of the dye molecule in Fig. 13. Notably, the absence of the parent molecular ion peak at *m*/*z* = 372 (crystal violet cation) or *m*/*z* = 408 (chloride form) confirms the effective degradation of the dye under solar light irradiation. Instead, several lower *m*/*z* fragment peaks were observed, indicating stepwise breakdown of the triphenylmethane structure. The peaks at *m*/*z* 341 and 305 correspond to sequential *N*-demethylation processes, where methyl groups are removed from the dimethylamino substituent by reactive oxygen species such as ˙OH and ˙O_2_^−^ radicals. Further degradation leads to cleavage of the central carbonphenyl bonds, producing diarylmethane-type intermediates at *m*/*z* 275 and 261. The most intense peak (base peak) at *m*/*z* 242 is attributed to a highly resonance-stabilized diphenylmethane carbocation, formed after the loss of one substituted phenyl ring. This indicates that the triphenylmethane backbone has been significantly disrupted. Subsequent fragmentation generates smaller aromatic amine derivatives, observed at *m*/*z* 212, 182, and 130, corresponding to substituted benzene type intermediates. The presence of very low *m*/*z* fragments at 104, 83, and 56 suggests further breakdown into small amine species, indicating advanced degradation stages approaching mineralization.^42^ Overall, the distribution of fragment ions confirms that crystal violet undergoes initial *N*-demethylation followed by cleavage of the triphenyl core and progressive formation of smaller aromatic and aliphatic amine intermediates, ultimately leading toward complete mineralization into CO_2_, H_2_O, and inorganic nitrogen species shown in Fig. 14.

## 5. Conclusion

The Sr–Bi_4_Ti_3_O_12_/Co_3_O_4_ heterojunction was successfully synthesized and confirmed by XRD, TEM, SEM, and XPS analyses, which revealed a well-defined heterostructure with uniform morphology, intimate interfacial contact, and enhanced surface area. UV-Vis spectroscopy indicated a reduced band gap of 1.6 eV, allowing strong visible-light absorption. Photocatalytic tests demonstrated that the heterojunction efficiently degraded nearly around 95% of crystal violet dye within 55 minutes, outperforming individual Bi_4_Ti_3_O_12_ and Co_3_O_4_ components. The enhanced activity is attributed to effective charge separation facilitated by the Sr-doping and the heterojunction interface, as evidenced by photoluminescence (PL). Furthermore, the catalyst retained over 62% of its activity after four consecutive cycles, highlighting excellent stability and reusability. These results confirm that Sr–Bi_4_Ti_3_O_12_/Co_3_O_4_ is a highly effective visible-light-driven photocatalyst with significant potential for environmental remediation applications.

## Author contributions

Rasmirekha Pattanaik: investigation, formal analysis, data curation, writing of the manuscript. Suresh Kumar Dash: conceptualization, supervision, writing – review and editing. Rishabh Kamal: formal analysis, data interpretation.

## Conflicts of interest

The authors have no competing interests to disclose.

## Supplementary Material

RA-OLF-D6RA03141B-s001

## Data Availability

The data supporting this article have been included as part of the supplementary information (SI). Supplementary information: the synthesis of both the photocatalysts and the reusuability procedure. See DOI: https://doi.org/10.1039/d6ra03141b.
