# Supplementary material for: Tailored Sr–Bi4Ti3O12/Co3O4 S-scheme heterojunction for highly efficient solar-light photodegradation of crystal violet dye
Source: RSC Adv. 2026 Jul 10;16(37):39570–86. doi: 10.1039/d6ra03141b (PMC13352666; doi:10.1039/d6ra03141b)
Supplement: Supplementary file 1 [file RA-016-D6RA03141B-s001.pdf]

This journal is © The Royal Society of Chemistry 2026

S1. Synthesis of 4 wt% Sr-$Bi_4Ti_3O_{12}$ (4 wt% Sr-BTO)

The 4 wt% Sr-$Bi_4Ti_3O_{12}$ (4 wt% Sr-BTO) photocatalyst was synthesized by a modified sol-gel method. Initially, 0.2M bismuth nitrate pentahydrate ($Bi(NO_3)_3 \cdot 5H_2O$) was taken in a 500 mL beaker and continuously stirred with an equal amount of 2M TiO_2(anatase phase) for 30 min. To obtain 4 wt% Sr doping, 0.093 g of strontium nitrate ($Sr(NO_3)_2$) was added to the precursor mixture under continuous stirring. The resulting suspension was sonicated for 10 min at room temperature to break down TiO_2 agglomerates and ensure homogeneous mixing. Subsequently, 0.4M oxalic acid solution was added dropwise under vigorous stirring, followed by the addition of 10 mL of 0.1M ammonia solution until the pH of the mixture reached approximately 6-7. The precipitate formed was repeatedly washed with deionized water, filtered, and dried at 40^0C for 8 h in a vacuum oven. The dried product was then calcined in a muffle furnace at 500^0C for 4 h with a heating rate of 2^0C min^{-1}. Finally, the obtained powder was ground using an agate mortar and pestle to obtain the 4 wt% Sr-$Bi_4Ti_3O_{12}$ (4 wt% Sr-BTO) photocatalyst.

18. R. Pattanaik, D. Pradhan, R. Kamal and S. K. Dash, *Next Materials*, 2025, **8**, 100847.

S2. Synthesis of Co_3O_4

Co3O4 nanoparticles were synthesized by a facile sol-gel method. Briefly, 100 mL of 0.1 M cobalt nitrate hexahydrate [$Co(NO_3)_2 \cdot 6H_2O$] solution was prepared in a 250 mL beaker and magnetically stirred at room temperature for 30 min to obtain a homogeneous solution. Subsequently, 50 mL of 0.2 M sodium hydroxide (NaOH) solution was added dropwise under vigorous stirring. The reaction mixture was then heated at 100^0C and maintained for an additional 30 min, leading to the formation of a pink-coloured gel.

The resulting gel was collected by centrifugation at 5000 rpm for 15 min and further filtered using a Buchner funnel. The obtained precipitate was dried in an oven at 100^0C for 3 h and subsequently calcined in a muffle furnace at 600^0C for 3 h to facilitate the formation of crystalline Co_3O_4 nanoparticles. Finally, the calcined product was gently ground using an agate mortar and pestle to obtain a fine powder for further characterization and photocatalytic studies.

The formation of Co_3O_4 proceeds via the initial precipitation of cobalt hydroxide followed by its oxidation during calcination, as represented by the following reactions:

$$Co^{2+} + 2OH^{-} \rightarrow Co(OH)_2 \quad (1)$$

$$3Co(OH)_2 + 2OH^{-} \rightarrow Co_3O_4 + 4H_2O + 2e^{-} \quad (2)$$

19. D. Pradhan, S. K. Biswal, R. Pattanaik, N. Nayak and S. K. Dash, *New Journal of Chemistry*, 2024, **48**, 16853–16868.

S3. Reusability Study of Sr-$Bi_4Ti_3O_{12}/Co_3O_4$ Photocatalyst

The reusability and stability of the synthesized Sr-$Bi_4Ti_3O_{12}/Co_3O_4$ heterojunction were evaluated through four consecutive photocatalytic degradation cycles of crystal violet (CV) dye under natural sunlight irradiation. In each cycle, 40 mg of photocatalyst was dispersed in 25 mL of 40 mg L^{-1} CV solution and irradiated under identical experimental conditions for 55 min.

At the end of each degradation experiment, the photocatalyst was recovered by centrifugation (5000 rpm for 10 min) and thoroughly washed several times with deionized water and ethanol to remove any adsorbed dye molecules and intermediate degradation products from the catalyst surface. The recovered material was then dried in a hot-air oven at 80 °C for 6 h and reused in the subsequent cycle without any additional treatment.

The degradation efficiency was calculated after each cycle to assess the recyclability of the photocatalyst. The catalyst exhibited excellent stability, retaining approximately 95% degradation efficiency during the first cycle and about 62% of its initial activity after four consecutive cycles. The slight decrease in photocatalytic performance may be attributed to the partial loss of catalyst during recovery and the accumulation of reaction intermediates on the catalyst surface. Nevertheless, the photocatalyst maintained considerable activity, demonstrating its good structural stability and potential applicability for repeated wastewater treatment operations.
